# NRXN1 deletions identified by array comparative genome hybridisation in a clinical case series – further understanding of the relevance of NRXN1 to neurodevelopmental disorders

**DOI:** 10.1186/2049-9256-1-4

**Published:** 2013-04-23

**Authors:** Sarah Curran, Joo Wook Ahn, Hannah Grayton, David A Collier, Caroline Mackie Ogilvie

**Affiliations:** 1 Department of Child and Adolescent Psychiatry, Institute of Psychiatry, Kings College London, De Crespigny Park, Denmark Hill, London, SE5 8AF UK; 2 Cytogenetics Department, Guy’s and St Thomas’ NHS Foundation Trust, London, UK; 3 MRC Social, Genetic and Developmental Psychiatry Centre, Institute of Psychiatry, King’s College London, De Crespigny Park, Denmark Hill, London, SE5 8AF UK

**Keywords:** Copy number variants, Autism spectrum disorders, NRXN1, Neurodevelopmental disorders, Epilepsy, Microcephaly, Neurexins

## Abstract

**Background:**

Microdeletions in the NRXN1 gene have been associated with a range of neurodevelopmental disorders, including autism spectrum disorders, schizophrenia, intellectual disability, speech and language delay, epilepsy and hypotonia.

**Results:**

In the present study we performed array CGH analysis on 10,397 individuals referred for diagnostic cytogenetic analysis, using a custom oligonucleotide array, which included 215 NRXN1 probes (median spacing 4.9 kb). We found 34 NRXN1 deletions (0.33% of referrals) ranging from 9 to 942 kb in size, of which 18 were exonic (0.17%). Three deletions affected exons also in the beta isoform of NRXN1. No duplications were found. Patients had a range of phenotypes including developmental delay, learning difficulties, attention deficit hyperactivity disorder (ADHD), autism, speech delay, social communication difficulties, epilepsy, behaviour problems and microcephaly. Five patients who had deletions in NRXN1 had a second CNV implicated in neurodevelopmental disorder: a CNTNAP2 and CSMD3 deletion in patients with exonic NRXN1 deletions, and a Williams-Beuren syndrome deletion and two 22q11.2 duplications in patients with intronic NRXN1 deletions.

**Conclusions:**

Exonic deletions in the NRXN1 gene, predominantly affecting the alpha isoform, were found in patients with a range of neurodevelopmental disorders referred for diagnostic cytogenetic analysis. The targeting of dense oligonucleotide probes to the NRXN1 locus on array comparative hybridisation platforms provides detailed characterisation of deletions in this gene, and is likely to add to understanding of the importance of NRXN1 in neural development.

## Background

Neurexin 1 (NRXN1; 2p16.3) is a member of a small family of proteins, which also includes neurexin 2 and neurexin 3, originally identified as synaptic transmembrane receptors for the black-widow spider toxin α-latrotoxin [1]. Neurexins play a role in synapse maturation by fine-tuning synaptic properties and regulating synaptic transmission through interaction with neuroligins [2] and mediate trans-synaptic interactions that help to shape the synapse [3]. Each neurexin gene (NRXN1-3) produces two major isoforms, α- and β-, with different extracellular but similar intracellular and transmembrane structures [4]. α-NRXNs, which act as synaptic organisers, have six LNS (L*aminin G*, N*RXN*, S*ex-hormone-binding globulin*) domains, with three intercalated epidermal growth factor (EGF)-like domains and have been shown to interact with neurexophilins [5] and LRRTM proteins [6], as well as regulating some calcium channels [7]; β-NRXNs have a single LNS domain, lack EGF-like sequences and contain fewer laminin G domains [8]. Nrxn1 also undergoes extensive alternative splicing, which is temporally and spatially controlled by neuronal activity via calcium/calmodulin-dependent kinase IV signalling [9].

Deletions within NRXN1 have been identified in individuals diagnosed with a range of neurodevelopmental disorders (NDD): including intellectual disability, developmental delay, speech and language delay [10, 11] autism spectrum disorders (ASD) [12–17] schizophrenia [18–21], and when homozygously deleted, early-onset epilepsy [22] or Pitt-Hopkins-like Syndrome [23]. A family with NRXN1 deletions, schizophrenia and type 1 diabetes has been described [24], which is plausible because neurexin 1 is expressed in β -cells of pancreas [25]. Although deletions in NRXN2 have not been reported, rare deletions in NRXN3 have been identified in ASD [26].

The above studies have mainly focused on the detection of CNVs in groups of patients with specific phenotypes. However, it is increasingly recognised that NRXN1 deletions may be risk factors for a variety of clinical disorders. In the present study, we report intragenic NRXN1 deletions detected during diagnostic cytogenetic testing of sequential referrals using a custom aCGH platform including dense coverage of the NRXN1 locus, and describe the phenotype of these patients and the size and position of their deletions.

## Materials

### Diagnostic referral cases

The tested cohort consisted of patients referred to Guy’s and St Thomas NHS Foundation Trust from regional paediatricians and other health specialists, as well as from genetics centres both in and outside the region (SE Thames). Array CGH analysis was initiated to determine the causes of developmental delay, neurocognitive disability, learning difficulties, behavioural abnormalities or birth defects or to confirm a clinical diagnosis of a suspected syndrome. All patient tests were carried out as part of standard clinical care, either as clinical referrals for array CGH testing following a normal karyotype, or those having array CGH as a first-line test in place of karyotyping. All data were anonymised.

### Array CGH analysis

Testing was carried out at a regional cytogenetics CPA accredited laboratory, according to published protocols [27], using an Agilent oligonucleotide 60 K array platform (designs 028469 and 017457) with a total imbalance detection rate of 24%. There are 215 NRXN1 probes on the array, with a median spacing of 4.9 kb.

Genomic data and referral phenotype information was recorded in a clinical database, which at the time of analysis contained 10,397 clinical referrals, including 1368 patients referred for ASD, 360 of whom were female. Copy number variants in this population are available in the Brain and Body Genetics Resource Exchange (BB-GRE; bbgre.org).

## Results and discussion

**Figure 1:**
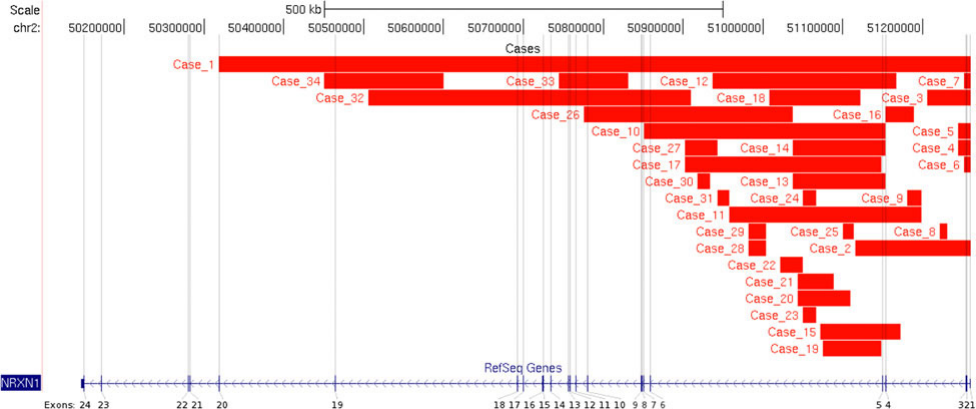
**Deletions in the NRXN1 gene (HG19) in the present series.** Exons are numbered according to refseq (Homo sapiens neurexin 1 (NRXN1), transcript variant alpha2, mRNA NCBI Reference Sequence: NM_001135659.1). Two of our patients have deletions that overlap with the beta isoform, patients 1, 34.

**Figure 2:**
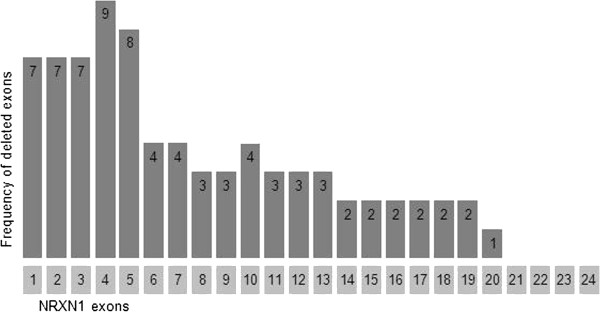
**Frequency of exonic deletions in the present series.**

We found 34/10,397 patients with deletions within NRXN1 (0.33% of referrals). The majority of these were within the region of the gene encoding the alpha isoform but two included exons which are part of one of the beta isoforms of NRXN1 (exons numbered 19–24 in the present study). Details of patients and deletions are shown in Table 1, and the positions of the deletions with respect to the exons are shown in Figure 1. The frequency of deletion of each exon is shown in Figure 2. Eighteen of the patients in the present study had at least one exon deleted (0.17%); the first three exons were deleted in seven patients, all with NDD. Patients 10–16 had deletions including exon 4, the most commonly deleted exon; five of these patients had NDD. Patient 16 had a deletion of exon 4 alone and had no noted neurodevelopmental problems, although the age at presentation (see Table 1) was such that neurobehavioural problems would not yet be evident. There is evidence that exon 4 is not expressed in murine brain tissue and therefore isoforms of NRXN1 including exon 4 may not impact on neurodevelopment [28]. We found deletions in more distal exons, from 6 to 19, in patients with developmental delay, autism or severe speech delay. The three patients with deletions of exons which also form part of the beta isoform of NRXN1 (patients 1 [Developmental delay], and 34 [Developmental delay], autism) did not have unusual referral phenotypes. As noted below, patient 34 also has a second potentially pathogenic CNV.

**Table 1:** **Deletions in the NRXN1 gene in clinical referrals for array CGH analysis in the present study**

Patient	Start (hg19)	Stop (hg19)	Size (kb)	Exons	Inheritance	Other imbalances	Referral indication	Age (yrs)
1	50,318,520	51,260,612	942	1-20	Paternal	-	Developmental delay	3
2	51,116,137	51,260,612	144	1-5	De novo	-	Developmental delay, failure to thrive, pulmonary stenosis, hearing disorder, microcephaly, short stature, ?Noonan syndrome	1
3	51,205,906	51,260,612	55	1-3	N.A.	-	Learning difficulties	9
4	51,245,074	51,260,612	16	1-3	N.A.	13q31.1(85,376,208-86,370,409)x1	ADHD, learning disability	41
5	51,245,074	51,260,612	16	1-3	N.A.	-	Developmental delay	4
6	51,251,498	51,260,612	9	1-3	N.A.	-	Delayed (atypical) cognitive development, speech & language development disorder, motor skills development disorder	1
7	51,251,498	51,260,612	9	1-3	N.A.	-	Marked developmental delay, marked hypotonia (generalised), hypoplastic nails	2
8	51,221,421	51,230,518	9	(intron 3)	N.A.	-	?Peutz-Jegher syndrome	11
9	51,180,561	51,199,026	18	(intron 3)	N.A.	-	Developmental delay, autism	2
10	50,850,691	51,153,106	302	4-7	N.A.	-	Developmental delay, speech delay	3
11	50,957,455	51,199,026	242	4-5	De novo	7q35(145,650,395-146,558,801)x1 pat (CNTNAP2)	Early-onset epilepsy, myoclonic seizures, speech delay	5
12	50,937,444	51,166,725	229	4-5	Maternal	14q21.1(41,234,592-41,532,307)x1 mat	Developmental delay	6
13	51,037,104	51,153,106	116	4-5	N.A.	-	Developmental delay, epilepsy, dystonia, microcephaly, squint	2
14	51,037,104	51,153,106	116	4-5	N.A.	-	Autism	7
15	51,072,302	51,172,182	100	4-5	N.A.	-	Amenorrhoea, premature ovarian failure	32
16	51,153,052	51,189,385	36	4	N.A.	-	Anterior anus	0
17	50,902,782	51,148,567	246	(intron 5)	Maternal	7q11.23(72,700,414-73,777,326)x1 (Williams Beuren syndrome)	Developmental delay, microcephaly, behaviour problems	4
18	51,008,023	51,122,150	114	(intron 5)	N.A.	-	Developmental delay, speech delay, small mouth, microcephaly	10
19	51,075,491	51,148,567	73	(intron 5)	N.A.	-	Congenital heart defect	0
20	51,043,498	51,109,749	66	(intron 5)	N.A.	-	Developmental delay, speech delay, social communication difficulties	3
21	51,043,498	51,088,201	45	(intron 5)	N.A.	-	Behavioural problems	6
22	51,021,452	51,049,704	28	(intron 5)	N.A.	-	Autism, moderate learning difficulties	11
23	51,049,645	51,066,637	17	(intron 5)	Maternal	-	Dysmorphic, ?bronchiolitis	0
24	51,049,645	51,066,637	17	(intron 5)	Maternal	-	Interuterine growth retardation, pitting oedema, duplex right kidney, undescended right testis, single palmar creases	0
25	51,100,412	51,113,311	13	(intron 5)	N.A.	-	Myoclonic epilepsy episodes since six weeks of age	0
27	50,902,782	50,943,419	41	(intron 5)	Paternal	-	Speech delay, social communication difficulties	5
28	50,982,113	51,003,663	22	(intron 5)	N.A.	22q11.21(18,896,971-21,377,825)x3 (22q11.2 duplication syndrome)	Autism	10
29	50,982,113	51,003,663	22	(intron 5)	N.A.		Dysplastic kidneys, ventral-septal defect,cryptorchidism	0
30	50,918,448	50,933,351	15	(intron 5)	Maternal	22q11.21(18,896,972-21,440,514)x3 dn (22q11.2 duplication syndrome)	Epilepsy with focal seizures	11
31	50,943,360	50,957,514	14	(intron 5)	N.A.	-	Learning difficulties, ADHD, autism	7
32	50,505,606	50,909,824	404	6-18	De novo	-	Developmental delay, severe speech delay	5
26	50,775,890	51,037,163	261	6-10	De novo	-	Developmental delay, hypotonia, speech delay	6
33	50,744,594	50,831,617	87	10-13	N.A.	-	Developmental delay	1
34	50,450,675	50,600,302	150	19	N.A.	8q23.3(113,960,008-114,131,155)x1 (CSMD3)	Developmental delay, autism	4

“Patient” refers to numbering in the text. “Inheritance” indicates if the imbalance was inherited (and if so from which parent, or de novo. N.A. is not assessed). Age (yrs) is age at testing.

NDDs were present in 27 out of the 34 patients, and included developmental delay, learning difficulties, ADHD, autism, speech delay, social communication difficulties, epilepsy, behaviour problems and microcephaly; of the remaining seven patients, five were below the age of 1 year, and would not necessarily be expected to present with such clinical features; follow-up studies on these children may provide further information, Sixteen of the patients had intronic deletions, two with deletions within intron 3 (one with NDD) and ten with intron 5 deletions (seven with NDD). Two of our subjects (19 and 29), both with deletions in intron 5, had cardiac defects. Three patients with intronic deletions had other, syndromic imbalances (Williams-Beuren syndrome (patient 17) and 22q11.2 duplication syndrome (patients 28 and 30)). Patient 11 (exons 4–5 deletion) also had a paternally inherited CNTNAP2 deletion and patient 34 (exon 19 deletion) had an intragenic deletion of CSMD3. Two other patients (4 and 12) carried additional copy number variants of uncertain significance.

Our present study describes a large series of NRXN1 deletions identified through a clinical genetics service. This data adds to the understanding of the genotype/phenotype correlation of NRXN1 deletions. Previously, Rujescu et al. [21] described the detection of twelve NRXN1 deletions in 2,977 patients with schizophrenia, of which seven (0.24% of the patient population) were exonic. These predominantly affected the 5’ exons of the gene, consistent with ablation of the alpha-isoform being the most common type of pathogenic mutation in NRXN1. They also found that ~ 0.14% of normal controls carried intronic NRXN1 deletions, and since this frequency did not differ significantly from that of intronic deletions in cases, they concluded that these were not likely to be pathogenic. Subsequently, Ching et al. [10] reported 12 exonic NRXN1 deletions in a group of 3,540 patients referred for array CGH testing for a variety of different phenotypes, giving a prevalence of 0.34% in this group. A further recent study [29] described 24 patients with intragenic deletions of NRXN1. Seventeen of these deletions involved exons of NRXN1, including four cases with involvement of exons encoding β-neurexin.

In the present study, 18 deletions in the case series were exonic (including two deletions which encompass both the alpha and beta isoforms of NRXN1), and the remainder intronic. The lower prevalence of exonic deletions in our study group (0.17%) than that of other studies [10, 20, 21] may reflect the wide range of referral indications in our cohort, and is very similar to the overall prevalence of 14/8798 (0.16%) calculated from combining totals from a number of published studies of schizophrenia [19].

The rate of intronic deletions we found in our case sample is similar to that seen by Rujescu et al. [21], and although approximately two thirds of our 13 patients with “intronic only” NRXN1 deletions had overt neurodisability phenotypes, this is most likely a result of the nature of the clinical referral population, as about two-thirds of referrals have this class of phenotype. For three of the patients with intronic NRXN1 deletions in our cohort, a second CNV was present (one Williams-Beuren deletion, and two 22q11.2 duplications), likely to have been contributory factors in their neurodisability.

However it is still possible that some intronic deletions in NRXN1 are pathogenic through the deletion of essential regulatory elements in the gene, such as alternative promoters, enhancers, or sequences involved in the complex splicing which generates various isoforms of NRXN1 mRNA. For example, Iijima et al., [9] found that alternative NRXN1 splicing (inclusion of the cassette exon 20; this is exon 21 using the refseq numbering in the present study) is dependent on the presence of AU-rich recognition sequences for SAM68 and related RNA-binding proteins in the flanking introns, and that the deletion of these has functional consequences. However we did not identify mutations encompassing the exon-21 introns in the present study.

There are many examples of pathogenic intronic deletions in the literature. For example intronic deletions in the SLC34A3 gene cause hereditary hypophosphatemic rickets with hypercalciuria [30], in the PKD1 gene cause Rothmund-Thomson syndrome [31], and in the dihydropyrimidine dehydrogenase gene cause 5-fluorouracil toxicity [32]. Several mechanisms that could account for this [33], including deletion of an unknown exon, deletion of an alternative promoter and effects on regulatory sequences such as those controlling splicing, causing intron retention, exon skipping and cryptic splice site activation through the deletion of cis-acting elements, or constraining the intron size to below that required for proper splicing [34].

In the present study, while we did not find deletions extending as far as the exon-21 flanking introns sequences, our finding of intron 5 deletions in 14 affected individuals is interesting. The potential importance of intron 5 deletions has previously been suggested by Ching and colleagues, who reported a de novo intron 5 deletion in an individual with PDD-NOS [10]. In order to show that intron 5 deletions are pathogenic, a case–control analysis and demonstration of the presence of functional sequence would be required.

We also attempted to determine how many of the NRXN1 mutations we found were de novo in origin. In only eleven of our cohort could inheritance be established; of these, four had apparently arisen de novo; however, the neurocognitive status of the carrier parents of the other seven patients was not known.

It has also become evident that a subset of patients with neurodevelopmental disorders may have more than one pathogenic mutation [35]. In the present study, patient 11 had a paternally inherited contactin-associated protein 2 (CNTNAP2) deletion in addition to a de novo deletion of exons 4 to 5 of NRXN1. CNTNAP2 is a member of the neurexin superfamily, and deletions of CNTNAP2 have also been associated with NDDs [11, 36]; it is therefore entirely consistent that compromise of both NRXN1 and CNTNAP2 would lead to a severe NDD, as seen in our case (early-onset epilepsy, myoclonic seizures and speech delay). Heterozygous deletions of either NRXN1 or CNTNAP2 have been found to be associated with severe intellectual disability [11]. Patient 34 had developmental delay and autism, and in addition to an exon 19 NRXN1 deletion, also carried a chromosome 8 deletion resulting in intragenic deletion of CSMD3, a gene whose function is currently unknown, but which has been shown to be expressed in adult and fetal brain [37]. Floris et al. [38] described two patients with autistic disorder and balanced translocations with breakpoints close to CSMD3 suggesting that this is a candidate gene for autism. Apart from infants less than 1 year old, only one patient (patient 15) had an exonic deletion with no reported neurodevelopmental problems: this patient was tested for amenorrhea and premature ovarian failure, and had a deletion of exons 4 to 5, suggesting that this specific deletion may not result in fully penetrant functional compromise of NRXN1.

Pleiotropy is also an important feature of most pathogenic copy number mutations [39]. NRXN1 has been associated with a wide range of disorders, including schizophrenia, autism, epilepsy, and intellectual disability. The reasons behind this phenotypic variability are unknown; however, the presence of a second hit - mutations in other genes in brain development pathways - is very likely to modulate the effects of NRXN1 deletions, and environmental factors may also play a part in the differential expression and penetrance of NDDs. Further insight into this process will be obtained from genome sequencing, which will be able to identify ‘second hits’ including point mutations and other rare variants.

## Conclusions

In the present study, we found a series of exonic deletions in the NRXN1 gene in patients referred for clinical diagnostic cytogenetic analysis. These deletions were found in patients with a range of neurodevelopmental disorders, including attention deficit hyperactivity disorder (ADHD), which until recently was not considered to have a genomic basis. NRXN1, like all neurexins, has two main isoforms, alpha and beta [4]. In common with other structural studies of NRXN1, our series of deletions predominantly affect the alpha isoform. Ullrich et al. [40] and Rowen et al. [41] have suggested that the presence of alternative promoters and the process of alternative splicing leads to the production of many different NRXN1 proteins. The structure and functional importance of these different proteins has not yet been elucidated, and further information on the phenotypic consequences, or lack thereof, of different NRXN1 deletions is therefore essential to the understanding of NDDs, and thence to the processes leading to normal brain development and function. Our data adds a further 18 exonic and 16 intronic deletions to the catalogue of previously published NRXN1 deletions. Interestingly, we found a high frequency of intron 5 deletions in our cohort; this finding may be worthy of further investigation.

## Abbreviations

NRXN1: Neurexin 1
NRXN2: Neurexin 2
NRXN3: Neurexin 3
aCGH: Array comparative genome hybridisation
HG19: The February 2009 human reference sequence (GRCh37/HG19).
